# Extracts from the Records of the Boston Society for Medical Improvement

**Published:** 1856-07

**Authors:** F. E. Oliver

**Affiliations:** Secretary


*Extracts from the Records of the Boston Society for Medical Improvement.* By F. E. OLIVER, M. D., Secretary.

APRIL 14th.—*Apoplexy during Labor. Effusion of Blood into the Pons Varolii and Crura Cerebri. Death.* Case reported by Dr. JOHN HOMANS.

Mrs. ———, aged 32 years, of plethoric habit and nervous temperament, though generally enjoying good health, with the exception of headache, to which she had been subject from childhood, was married April 9th, 1853. She became pregnant in April of the following year, and aborted in July, about the end of the third month. She again aborted at the same period of pregnancy in June, 1855. Her third pregnancy commenced immediately after this occurrence. She suffered much from sympathetic affection of the stomach during the whole of the first two pregnancies, and until the sixth month of the last. At about the middle of the eighth month, her limbs began to swell, and her whole body became gradually anasarcous, so that she was quite clumsy in her movements. Occasionally she complained of headache, especially in the morning. On the 20th of March, 1856, Dr. H. was called to see her on account of these two symptoms. About sixteen ounces of blood were taken from her, and some laxative medicine was ordered, together with a strictly farinaceous diet. The urine examined at this time, was found to be highly albuminous. Relief from her troublesome feelings followed this treatment until March 27th, when the headache returned, and the swelling, which had diminished, again increased. Blood-letting was again resorted to, to the amount of twelve ounces, and a cathartic given. The pain in the head ceased immediately after the bleeding; she expressed herself as feeling remarkably well, and engaged in the amusements of the family during the day and evening. About 1 o'clock, A. M., March 28th, the cathartic operated freely, after which she complained of nausea, and vomited a frothy mucus. This occurred several times, and was attended with severe pain in the epigastric region, which became so intense as to cause her mother to send for Dr. Homans at 3, A. M., though against the wishes of the patient, who thought the pain would soon pass off without medical aid. Dr. H. found her suffering from pain as above, and also in the head. Immediately after his arrival she had a severe convulsion, lasting three minutes; from this she rallied, and said she felt better. An hour after, a second convulsion occurred, more severe and of longer duration. She did not recover as before, and exhibited but slight indications of consciousness, which soon entirely disappeared. Her face during the convulsions became exceedingly livid, and this continued to be the case in some degree afterwards. She was perfectly motionless after this second attack; her eyes were shut, and her respiration labored though not stertorous. After an interval of two hours a third convulsion took place, less severe and shorter in duration than the others. At this time, about 8, A. M., the waters were spontaneously discharged, she having before exhibited some indications of being in labor, by pain, and bloody discharge from the vagina. The *os uteri* was dilated to about the size of a dime. The respiration soon became somewhat stertorous, though not at any time remarkably so. The pulse, before the first convulsion, was about 100; subsequently, between 80 and 90. Whenever there were symptoms of the commencement of a convulsion, sulphuric ether was administered by inhalation, with the apparent effect of averting them. She remained motionless and senseless, without any other convulsion, till death took place, about 1 o'clock P. M., suddenly and easily. There were no signs of the life of the fœtus after the first attack. The labor advanced no farther than above described.

*Autopsy*, 46 hours after death—the body having been preserved in ice.

*Head.* The vessels were well filled with blood; the convolutions somewhat flattened; considerable white softening of the *septum lucidum* and of the parts immediately surrounding the lateral ventricles, which contained much more fluid than usual. There was quite a large effusion of blood into the *pons varolii* and *crura cerebri.*

*Thorax.* The *lungs* and *heart* were normal.

*Abdomen.* Scattered through the substance of the *liver*, which was of a deep-yellow color, were a large number of dark-red maculæ, from half a line to one-fourth of an inch in diameter, resembling those of purpura on the surface of the body. The cortical substance of the *kidneys* had a somewhat rough, unhealthy look, and did not as strongly contrast with the tubular portions as in the majority of cases. On microscopic examination, nothing remarkable was noticed.

The *uterus* contained a well-formed female fœtus of the full term.

The other organs presented nothing remarkable.

The specimen consisted of the *pons varolii*, and portions of the cerebral matter immediately surrounding it. It has been preserved for more than two weeks by Dr. Putnam, in chloroform, and was quite unchanged, save that the consistence was somewhat firmer than at the autopsy. The clot was directly in the centre of the *pons varolii*, also involving the

*crura cerebri*, consisting of about half an ounce of blood. The portions of the brain in the immediate vicinity were somewhat softened and slightly yellow.—*Boston Medical and Surgical Journal.*

